# Impact of COVID-19 on Canadian Radiology Residency Training Programs

**DOI:** 10.1177/0846537120933215

**Published:** 2020-06-11

**Authors:** Devang Odedra, Baljot S. Chahal, Michael N. Patlas

**Affiliations:** 1 Department of Radiology, McMaster University, Hamilton, Ontario, Canada; 2 Department of Radiology and Diagnostic Imaging, University of Alberta, WC Mackenzie Health Sciences Centre, Edmonton, Alberta, Canada

**Keywords:** medical education, COVID-19, pandemic, emergency preparedness, virtual education

## Abstract

**Purpose:**

The novel coronavirus disease (COVID-19) pandemic has swept the globe, with a domino effect on medical education and training. In this study, we surveyed Canadian radiology residents to understand the impact of the pandemic on their residency training, strategies utilized by the residency programs in mitigating those impacts, and factors important to residents in the selection of educational resources on COVID-19.

**Methods:**

A 10-item questionnaire was distributed to 460 resident members of the Canadian Association of Radiologists. The survey was open for 2 weeks, with a reminder sent at half-way mark.

**Results:**

We received 96 responses (response rate: 20.9%). The 4 highest affected domains of training were daytime case volumes (92.4%), daytime schedules (87.4%), internal and external assessments (86.5%), and vacation/travel (83.3%). Virtual teaching rounds (91.7%), change in schedules to allow staying home (78.1%), and virtual/phone readouts (72.9%) were the most utilized strategies by the Canadian radiology residency programs. Overall stress of exposure to the disease was moderate to low (86.5%). A minority of the residents were redeployed (6.2%), although most (68.8%) were on standby for redeployment. Residents preferred published society guidelines (92.3%), review papers (79.3%), video lectures (79.3%), and web tools (76.9%) for learning about COVID-19 imaging manifestations.

**Conclusion:**

The COVID-19 pandemic has had a significant impact on various domains of the Canadian radiology residency programs, which has been mitigated by several strategies employed by the training programs.

## Abstract (fr)

**Objectif:**

La pandémie due au nouveau coronavirus (COVID-19) a balayé le globe, entraînant un effet domino sur l’éducation et la formation médicales. Dans le cadre de cette étude, nous avons interrogé des résidents canadiens en radiologie afin de comprendre l’incidence de la pandémie sur leur résidence en radiologie, les stratégies utilisées par les programmes de résidence pour atténuer ces répercussions, et les facteurs importants à leurs yeux pour la sélection des ressources éducatives sur la COVID-19.

**Méthodes:**

Nous avons distribué un questionnaire de 10 questions à 460 résidents membres de l’Association canadienne des radiologistes. Le questionnaire a circulé pendant deux semaines, avec un rappel envoyé à mi-parcours.

**Résultats:**

Nous avons reçu 96 réponses (taux de réponse : 20,9 %). Les 4 éléments de formation les plus touchés sont le nombre de cas vus dans la journée (92,4 %), les horaires de jour (87,4 %), les évaluations internes et externes (86,5 %) et les vacances/voyages (83,3 %). Les rondes de formation virtuelle (91,7 %), la modification des horaires pour permettre de rester chez soi (78,1 %) et les lectures virtuelles/au téléphone (72,9 %) ont été les stratégies les plus utilisées par les programmes canadiens de résidence en radiologie. Le stress global en lien avec l’exposition à la maladie était de modéré à faible (86,5 %). Une minorité de résidents a été réaffectée (6,2 %), bien que la plupart (68,8 %) aient été en attente de réaffectation. En ce qui concerne l’information sur les manifestations de la COVID-19 dans le domaine de l’imagerie, les résidents ont préféré les directives publiées par les sociétés de radiologie (92,3 %), les articles de synthèse (79,3 %), les conférences vidéo (79,3 %) et les outils en ligne (76,9 %).

**Conclusion:**

La pandémie de COVID-19 a eu des répercussions importantes sur divers éléments des programmes canadiens de résidence en radiologie, une incidence qui a été atténuée au moyen de plusieurs stratégies employées par les programmes de formation.

## Introduction

The novel coronavirus disease (COVID-19) pandemic has rapidly swept across the globe, causing unprecedented impact on the health system.^1^ The pandemic led to significant alterations and disruptions in the Canadian system,^2^ with a domino effect on medical education and training.

The physical distancing rules and safety precautions led to restructuring of radiology department workflow to minimize physical presence at work, preventing in-person case reviews and teaching sessions.^3^ The majority of elective medical procedures and imaging studies were put on hold, leading to drastic reduction in case volumes. Certain provincial health systems mandated redeployment of their trainees to areas other than their core training to secure adequate manpower for caring of current and projected cases of COVID-19.^4^ Numerous medical conferences and examinations were either cancelled or postponed, creating barriers in knowledge dissemination and licensure accreditation.^5,6^ The well-being and safety of trainees became increasingly important as they continued to provide daytime and on-call coverage during the pandemic. Radiology trainees also needed to educate themselves on the spectrum of imaging manifestations of COVID-19 in a relatively short period of time, given that they would likely encounter COVID-19 patients during their daytime and on-call coverage.

In this study, we aimed to survey Canadian radiology residents on the impact of COVID-19 on their residency training by means of a web-based questionnaire. We also included questions to better understand the factors important to trainees in their choice of educational resources on the imaging manifestations of COVID-19. We hoped that the results of this resident-based questionnaire would shed light on the drastic impact of the pandemic on clinical and educational training, provide ideas for improvement in radiology education, and help us understand the factors important to residents in their selection of educational resources.

## Methods

The project was approved by the Hamilton Integrated Research Ethics Board and the Canadian Association of Radiologists (CAR). A web-based 10-item questionnaire on the SurveyMonkey (SVMK Inc) platform was designed, which included a combination of multiple-choice, rating, and text-based questions (Online Appendix 1). Two of the coauthors (D.O. and B.C.) are radiology residents and involved in the CAR Resident and Fellow Section at the time of writing this manuscript. Their roles assisted them in the design of the questionnaire.

The CAR assisted our project by contacting all 460 resident members who previously agreed to receive promotional emails on May 1, 2020. Willing members were asked to complete an electronic survey via a link within the email. The survey remained open for 2 weeks, until May 15, 2020. A reminder email was sent at approximately the halfway mark to all recipients. To optimize survey integrity, the online survey platform was structured to permit only 1 response per computer IP address. Clicking on the link and submitting the survey responses implied consent to participate. Other than basic demographic information, no individually identifying participant data were collected. Upon closure of the survey, the responses were tabulated and plotted in Microsoft Excel.

## Results

### Demographics

**Figure 1.**
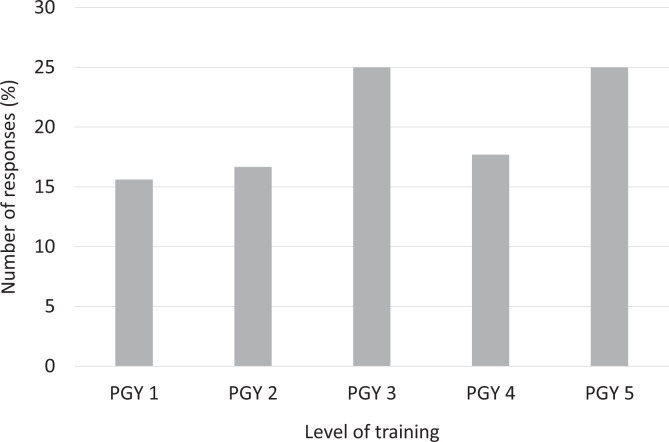
Breakdown of responses by the level of training. PGY indicates postgraduate year.

**Figure 2.**
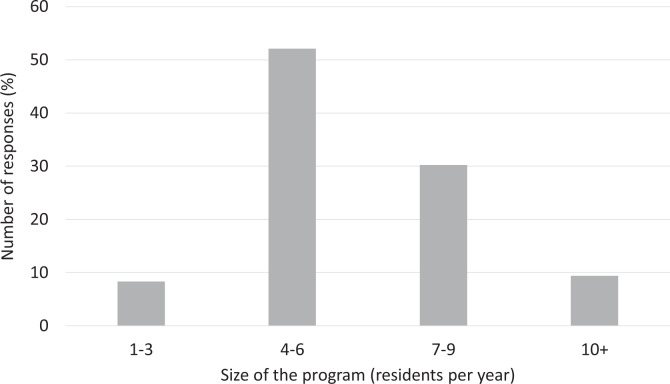
Breakdown of responses by the size of the program.

**Figure 3.**
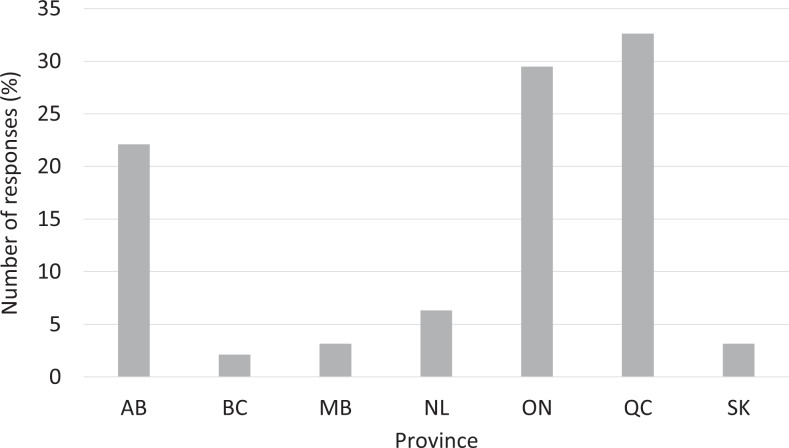
Breakdown of responses by the province. AB indicates Alberta; BC, British Columbia; MB, Manitoba; NL, Newfoundland and Labrador; ON, Ontario; QC, Quebec; SK, Saskatchewan.

A total of 96 recipients responded to the questionnaire (response rate of 20.9%). The proportion of respondents in each year of training ranged from 15.6% to 25.0% (Figure 1). More than half of the respondents were in a moderate-sized training program with 4 to 6 residents per year (Figure 2). Quebec, Ontario, and Alberta had the 3 highest number of respondents (32.6%, 29.5%, and 22.1%, respectively; Figure 3).

### Impact of COVID-19 on Training

**Figure 4.**
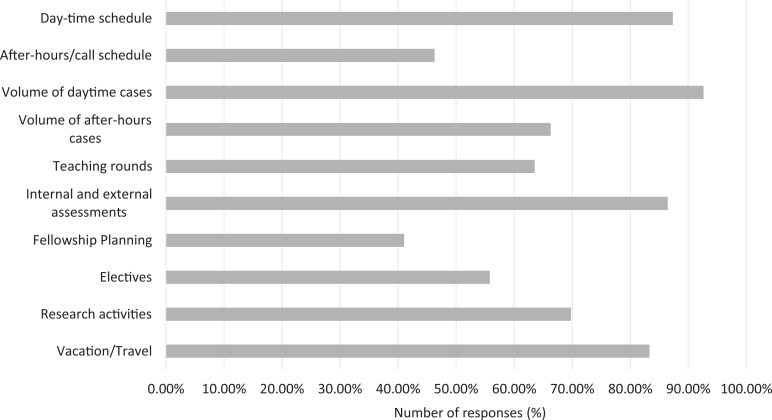
Breakdown of responses by the impact on the residency domain. Number of responses represents the portion of responses selecting “moderate” or higher on question 4.

Nearly all domains of residency training were impacted by the pandemic (Figure 4), albeit to varying degrees. Daytime schedule was more affected compared to after-hours, as evident by 87.4% of respondents selecting “moderate” or higher level for the disruption in daytime schedules, compared to 46.3% for the after-hour schedules. A similar trend was also observed for the volume of cases, where 92.6% of respondents selected “moderate” or higher level for the daytime volumes, as compared to 66.3% for the nighttime volumes. There was a variable impact on teaching rounds, with 19.8% having “moderate,” 26.0% having “a lot,” and 17.7% having “a great deal” of impact. Up to 86.5% of the respondents experienced “moderate” or higher level of disruption of internal or external assessments. Fellowship planning was relatively less impacted, with 41.1% selecting “moderate” or higher level. Similarly, electives were not as significantly impacted, with 55.8% of respondents selecting “moderate” or higher level. For the disruption of research activities, 69.8% of respondents selected “moderate” or higher level of disruption. Finally, vacation and travel plans were significantly affected, with 83.3% of respondents selecting “moderate” or higher level.

### Stress and Anxiety Levels

**Figure 5.**
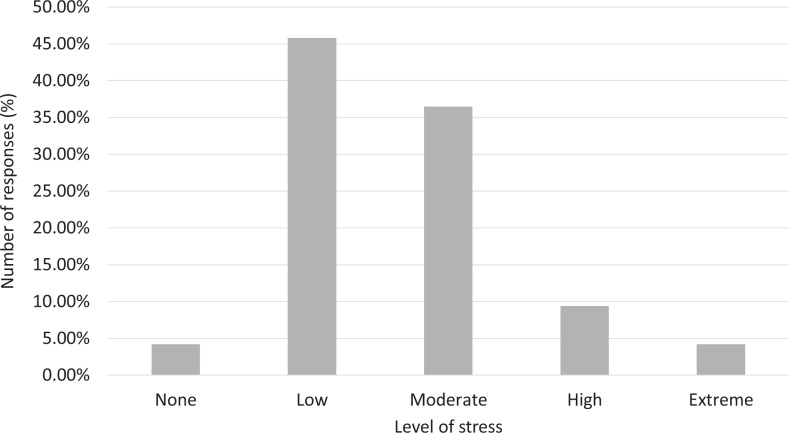
Breakdown of responses by the level of stress regarding exposure to the disease.

The level of stress and anxiety related to work-related exposure to COVID-19 was overall moderate to low, with 86.5% of responses in “moderate” or lower categories (Figure 5).

### Redeployment

**Figure 6.**
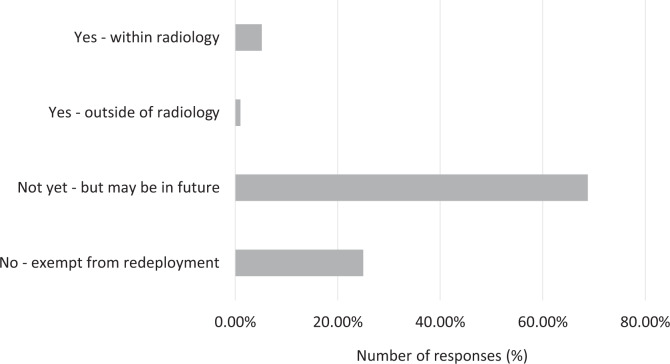
Breakdown of responses by the redeployment status.

Only a minority of residents were redeployed, with 5.2% being redeployed to a different service within radiology and 1.0% outside radiology (Figure 6). Up to 68.8% of respondents were not yet redeployed but were on standby for possible redeployment in the future, and 25.0% of respondents were exempt from redeployment.

### Strategies Utilized by the Programs

**Figure 7.**
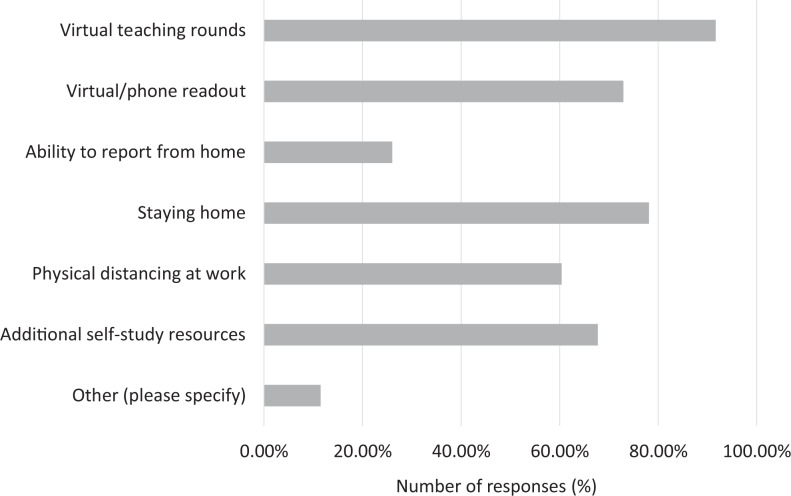
Breakdown of responses by the mitigation strategies utilized by the training programs.

A large number of respondents’ programs utilized virtual teaching rounds (91.7%), virtual/phone case readouts (72.9%), change in schedules to allow either staying home (78.1%) or maintaining physical distancing (60.4%), and offering of additional educational resources (67.7%; Figure 7). A small number of respondents (26.0%) had the ability to report from home. Up to 11.5% of respondents selected “other” strategies, which were provided via a text-based input box. Some of these comments are provided in Online Appendix 2.

### Web Conference Tools

**Figure 8.**
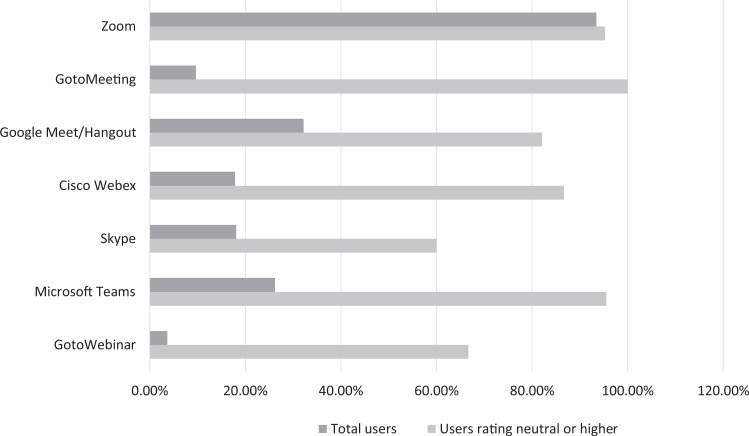
Breakdown of responses by the use and rating of virtual conference software.

The most commonly utilized application was Zoom (Zoom Video Communications Inc; 93.5%), followed by Google Hangout/Meet (Google LLC; 32.2%), Microsoft Teams (Microsoft Corporation, Redmond, California; 26.2%), Skype (Skype Technologies; 18.1%), Cisco Webex (Cisco Webex; 17.9%), GotoMeeting (LogMeIn Inc; 9.6%), and GotoWebinar (LogMeIn Inc; 3.7%; Figure 8). The satisfaction level for the 3 most utilized applications marked as “neutral” or higher was 95.3% for Zoom, 82.1% for Google Hangout/Meet, and 95.5% for Microsoft Teams.

### Resident Selection of COVID-19 Educational Resources

**Figure 9.**
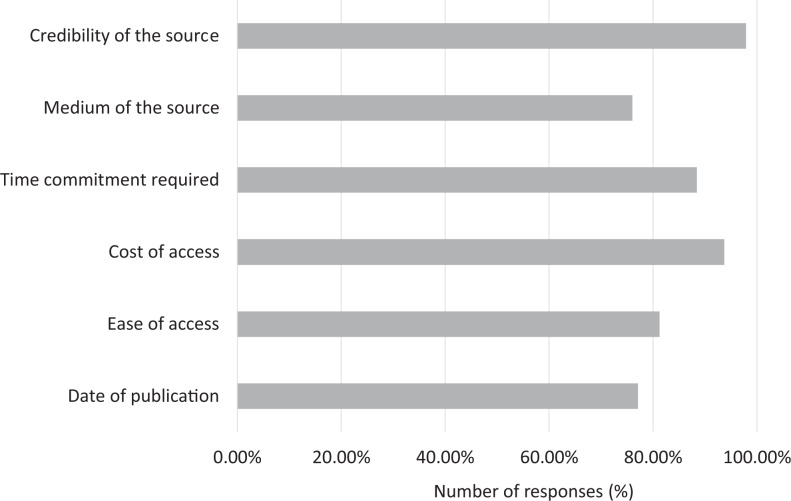
Breakdown of responses by the importance of factors in the selection of educational resources on imaging manifestations of COVID-19. Number of responses represents the portion of responses selecting “moderate” or higher on question 9.

When it came to self-education about the imaging manifestations of COVID-19, the top 3 factors were credibility of the source, cost of access, and time commitment required (97.9%, 93.8%, and 88.4%, respectively, selecting “important” or higher categories; Figure 9). These were followed by the ease of access (81.3%), date of publication (77.1%), and medium of the source (76.0%).

**Figure 10.**
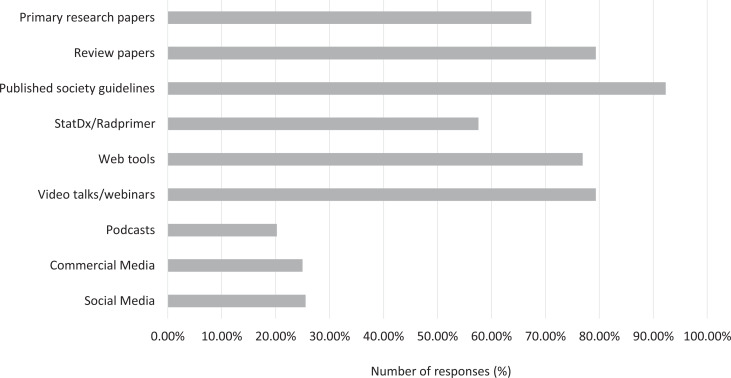
Breakdown of responses by the preferred educational resource type. Number of responses represents the portion of responses selecting “moderate” or higher on question 10.

In terms of the type of resource, a large number of residents ranked published society guidelines as “moderate” or higher (92.3%; Figure 10). This was followed by video talks/webinars, review papers, web-based tools, primary research papers, and StatDx/Radprimer (79.4%, 79.3%, 76.9%, 67.4%, and 57.6%, respectively). Social media, commercial media, and podcasts were overall ranked low (25.6%, 25.0%, and 20.2%, respectively). In terms of lack of access, podcasts and StatDx/Radprimer had the highest responses (16.9% and 12.0%, respectively).

## Discussion

The COVID-19 pandemic quickly swept the world in the spring of 2020. Canada crossed the 100-case mark on March 10, 2020, with rapid increase in the cumulative case count over the next 2 months reaching 79 411 and total number of deaths reaching 5960 by May 18, 2020.^7^ Although the rate of new cases has slowed down at the time of writing this manuscript, the pandemic is far from over. In this study, we aimed to poll Canadian radiology residents amid the pandemic at a point when the impact on their training was likely at its peak. Radiology medical educators across North America promptly recognized and anticipated the significant impact of the pandemic on radiology training. Several pieces of guiding principles and framework aimed at mitigating the impact were published in a timely manner.^3,4,8-10^ However, at the time of writing this manuscript, no direct questionnaire-based comparisons are available within radiology. Similar studies have been conducted in other specialties,^11^ and we anticipate that the radiology community will be conducting similar studies in the days to come.

In our study, we received a response rate of 20.9% (96/460), which is comparable to prior studies that surveyed a similar population in Canada.^12,13^ All the residency levels were relatively well represented, ranging from 15.6% to 25.0%. As expected, most of the responses were from moderate-sized programs (4-6 or 7-9 residents per year), as these programs overall dominate in Canada. Responses from Quebec, Ontario, and Alberta comprised 84.2% of all responses, which is not surprising since these provinces have multiple training institutions.

Respondents experienced an overall higher disruption in daytime schedules and case volumes compared to after-hour volumes, likely as elective and nonemergent studies, which were primarily affected by the pandemic and almost exclusively take place during the daytime. Joint statements were released at the outset of the pandemic by the CAR, Canadian Society on Thoracic Radiology, Canadian Association for Interventional Radiology, and Canadian Society of Breast Imaging, with a common theme of rescheduling nonemergent imaging and procedures.^2,14,15^ Teaching rounds were moderately affected, perhaps as they were conducted virtually rather than being cancelled. Internal and external assessments, which may include program Objective Structured Clinical Examination, the Medical Council of Canada Qualifying Examination (MCCQE), or the Royal College of Physicians and Surgeons of Canada Examination, were heavily affected. The MCCQE and the Royal College licensing examinations were eventually postponed with a modified format.^16,17^ Respondents were not as affected in terms of fellowship planning, likely because during the spring period, residents either have already gone through the previous application cycle or are working on the next cycle. Conducting virtual interviews for fellowship has been proposed if the pandemic continues into the interview cycle.^4^ Electives were also relatively less affected, which is likely because radiology is not an elective heavy training program and the majority of electives are usually completed within the resident’s home program. Vacation and travel plans were significantly affected as widespread travel advisories and transportation cancellations took place.^18^

Redeployment is a well-recognized consequence in the later stages of the pandemic when manpower needs to be optimally mobilized to care for COVID-19 cases.^9^ Fortunately, this affected only 6.4% of respondents, although up to 68.1% of residents were on standby for redeployment.

The impact on the psychological well-being of the trainees during the pandemic has also been identified with many helpful resources outlined in the literature.^3,4,9,19^ In our questionnaire, the overall stress level associated with exposure to COVID-19 was low. This is likely due to the strategies utilized by the programs in minimizing time at work and allowing physical distancing, as well as the fact that radiology is primarily a non-patient-facing specialty.^20^

The programs employed a variety of strategies to mitigate the impact of the pandemic, many of which have been outlined in the recent literature.^3,4,8-10^ As expected, the most widely utilized approach was the adoption of virtual modalities for providing teaching rounds and case reviews. Many residency programs around the world resorted to virtual modalities for delivering educational programming to their trainees. Additionally, a large number of respondents’ programs ensured physical distancing by modifying daytime and after-hour schedules. This is in line with the strategy outlined by Chong et al^4^ where at some of the California-based programs, 2 separate teams were created in terms of onsite and off-site duties. Many respondents also reported additional educational resources offered by their programs. This is in keeping with the overall spirit of the radiology community in openly embracing the challenges of the pandemic and providing numerous virtual educational opportunities to trainees.

Given the heavy reliance on web-conferencing tools for the virtual education of radiology trainees, we felt it was important to poll the trainees in terms of the software they have utilized and their overall satisfaction with the experience. Zoom was, by far, the most utilized software (93.5%), with Google Meet/Hangout (32.9%) and Microsoft Teams (26.8%) as the distant second and third. The respondents were overall satisfied with their experience on Zoom (95.3% rating it neutral or higher). We would like to direct the readers to 2 excellent summaries recently published by Lewis et al^21^ and Li et al^22^ on web conferencing tools relevant to radiology.

There has been a rapid influx of medical literature in various forms on the topic of COVID-19, including a myriad of studies and resources on the spectrum of multimodality imaging features of COVID-19.^23-26^ This includes primary papers, review papers, society guidelines, webinars/video lectures, and web tools.^27-29^ To this end, we found that residents value the credibility of the source, the cost of access, and the required time commitment the most. This is not surprising as residents experience increasing financial stressors and pressures of time management.^3^

In terms of the type of educational resource, overall trend was toward summary-type resources. Published society guidelines were the highest ranked (92.3%). We suspect this is because guidelines are a culmination of primary research and expert opinions, providing a reliable form of information. Similarly, review papers were more favored than primary papers (79.3% vs 67.4%). Video lectures/webinars and web tools were also moderately favored with 79.4% and 76.9%, respectively. Although recent work has been conducted on study habits and choice of educational resources in other specialties,^30,31^ the literature is sparse in radiology.^32^ Our study certainly opens an avenue for further research on this topic.

This study is not without limitations. A higher response rate to the questionnaire would have certainly benefited the study. We did optimize the questionnaire to ensure a high response rate, including limiting the number of questions, predominantly having multiple choice format and sending reminder emails.^33^ One of the major barriers to questionnaire completion may have been a higher-than-usual number of questionnaires circulating through the system currently amid the ongoing pandemic leading to “survey fatigue.” Additionally, some of the presumed recipients may not have enabled communication from CAR, although we suspect this number is likely small. There was also a disproportionally higher number of responses from Quebec, Ontario, and Alberta, which also happen to be the provinces most affected by the pandemic, causing a selection bias. Our questionnaire pushed the recipients to choose from a list of options, which may cause a response bias. We mitigated this by allowing open-text answers to some of the questions (Online Appendix 2).

We believe that our study has a strong practical value for medical education. First, it sheds light on the impact of the pandemic on various domains of radiology training, which will help medical educators in revising curricula in the short term (eg, making up for the lost exposure and time to certain areas). Second, it provides insight into some of the strategies that were utilized by the training programs across the country, which will assist in planning for the current pandemic peak as well as any possible future waves. Third, our questionnaire provides an idea of the residents’ experience with web conferencing software, which will be helpful in future as the role of virtual interaction will likely increase, even beyond the pandemic. A timely example of this is the recent decision to conduct interviews virtually for the next cohort of Canadian residency applicants.^34^ Lastly, the feedback from the residents on their choices of educational material will help the educators focus on what is important to the residents (eg, credibility of the source, cost of access) and design effective educational tools (eg, society guidelines, review papers, video lectures, web-based tools).

In conclusion, the COVID-19 pandemic has had a significant impact on various domains of the Canadian radiology residency programs, which has been mitigated by several strategies utilized by the training programs. The credibility of the source, cost of access, and time commitment required were important factors to residents for self-education on COVID-19. Additionally, summary-type resources were favored by the residents.

## Supplemental Material

Supplemental Material, Appendix_1 - Impact of COVID-19 on Canadian Radiology Residency Training Programs Click here for additional data file. Supplemental Material, Appendix_1 for Impact of COVID-19 on Canadian Radiology Residency Training Programs by Devang Odedra, Baljot S. Chahal and Michael N. Patlas in Canadian Association of Radiologists Journal

## Supplemental Material

Supplemental Material, Appendix_2_MP - Impact of COVID-19 on Canadian Radiology Residency Training Programs Click here for additional data file. Supplemental Material, Appendix_2_MP for Impact of COVID-19 on Canadian Radiology Residency Training Programs by Devang Odedra, Baljot S. Chahal and Michael N. Patlas in Canadian Association of Radiologists Journal
