# Supplementary material for: Impact of COVID-19 on Canadian Radiology Residency Training Programs
Source: Can Assoc Radiol J. 2020 Jun 11:0846537120933215. doi: 10.1177/0846537120933215 (PMC7290107; doi:10.1177/0846537120933215)
Supplement: Supplemental Material, Appendix_1 - Impact of COVID-19 on Canadian Radiology Residency Training Programs [file Appendix_1.pdf]

PARTICIPANT INFORMATION

1) Level of training – multiple choice

   PGY1 PGY2 PGY3 PGY4 PGY5

2) Size of the program in terms of residents per year - multiple choice

   1-3 4-6 7-9 10+

3) Province of training – dropdown list

   AB BC MB NL NS ON QC SK

IMPACT OF COVID-19 ON TRAINING

4) Rate the level of disruption due to COVID-19 on each of the following aspects of your training. (rating scale)
   a. Day-time schedule
   b. After-hour/call schedule
   c. Volume of daytime cases
   d. Volume of after-hours cases
   e. Teaching rounds
   f. Disruption of internal and external assessments (e.g. departmental OSCEs, MCCQE and Royal College Exams)
   g. Fellowship planning
   h. Electives
   i. Research activities (e.g. conferences)
   j. Vacation/Travel
   k. Other (please specify)

   Following options for each:
   None at all
   Little bit
   Moderate
   A lot
   A great deal

5) Have you been redeployed? (multiple choice)
   a. Yes – within radiology department
   b. Yes – outside of radiology department
   c. Not yet – but may be redeployed in future
   d. No – exempt from redeployment

6) What is your level of stress/anxiety regarding work-related COVID-19 exposure? (rating)

   None Low Moderate High Extreme

7) What did your program utilize to circumvent these? Select all that apply (multiple choice)
    a. Virtual teaching rounds
    b. Virtual/phone readout
    c. Ability to report from home
    d. Change in schedules to allow staying home
    e. Change in schedules to allow social distancing at work
    f. Offering additional self-study resources
    g. Other (please specify)

8) Please select which of the following web conference software you have come across during this pandemic and your level of satisfaction with each. (rating scale)
    a. Zoom
    b. GotoMeeting
    c. Google Meet/Hanout
    d. Cisco Webex
    e. Skype
    f. Microsoft Teams
    g. GotoWebinar
    h. Other

Following choices for each:
Did not use
Very dissatisfied
Somewhat dissatisfied
Neutral
Somewhat satisfied
Very satisfied

<u>COVID-19 PREPAREDNESS – KNOWLEDGE ACQUISITION</u>

9) How important is each of following factors when you are choosing educational material on COVID-19? (Rating scale)
    a. Credibility of the source
    b. Medium of the source (podcast, video, etc.)
    c. Time commitment required
    d. Cost of access
    e. Ease of access (e.g. readable on mobile device)
    f. Date of publication

Following choices for each:
Not at all important
Slightly important
Important
Fairly important
Very important

10) Rank each of the following educational resources in terms of their value for radiological manifestations of COVID-19 (ranking). Please select N/A if you do not have access to it. (rating scale)

    a. Primary research papers
    b. Review papers
    c. Published society guidelines
    d. Web tools (eg. researchassistant.nl)
    e. Video talks/webinars
    f. Podcasts
    g. Commercial Media (news, articles)
    h. Social Media
    i. StatDx/Radprimer

    Following options for each:
    None at all
    Little bit
    Moderate
    A lot
    A great deal
    N/A
