# Supplementary material for: Impact of COVID-19 on Canadian Radiology Residency Training Programs
Source: Can Assoc Radiol J. 2020 Jun 11:0846537120933215. doi: 10.1177/0846537120933215 (PMC7290107; doi:10.1177/0846537120933215)
Supplement: Supplemental Material, Appendix_2_MP - Impact of COVID-19 on Canadian Radiology Residency Training Programs [file Appendix_2_MP.pdf]

Text-based responses under the Other (please specify) answer choice for Question 7 (See Appendix 1 for the questionnaire):

"Removing every residents from the hospital, no more clinical exposition. We have to prepare course that they will use in the future instead of making their own teaching's material"

"Pedagogical work from home. No teaching. More work on call (all x-ray have to be read)."

"Numerous exposure to virtual teaching, self study, resources etc"

"Cancel rotations and move to ER coverage from a central site to minimize travel between sites"

"Incredible moral support"

"Backup call schedules created, weekly RPC meetings to actively address issues"

"Our program provides 1-2 hours of daily credits for wellness activities."

"junior residents no longer work during daytime"
